# Therapeutic potential of *Glycyrrhiza* polysaccharides in pseudorabies virus infection: immune modulation, antioxidant activity, and gut microbiota restoration

**DOI:** 10.3389/fvets.2025.1679013

**Published:** 2025-10-17

**Authors:** Chunlian Song, Qianfei Wei, Hong Shen, Xue Zhang, Deng Pan, Zhihui Zhang, Ying Zhang, Shanhai Yang, Xianghua Shu

**Affiliations:** ^1^The Yunnan Key Laboratory of Veterinary Etiological Biology, College of Veterinary Medicine, Yunnan Agricultural University, Kunming, China; ^2^Tacheng Animal Husbandry and Veterinary Station, Tacheng, China; ^3^Mangshi Animal Husbandry Station, Mangshi, China

**Keywords:** *Glycyrrhiza* polysaccharides, pseudorabies virus, gut microbiota, intestinal barrier, oxidative stress, immune regulation

## Abstract

**Aim of the study:**

This study aimed to evaluate the protective effects of *Glycyrrhiza* polysaccharides (GPs) on Pseudorabies virus (PRV)-infected mice and elucidate their mechanisms of action, with a focus on intestinal immunity, oxidative stress, mucosal barrier function, and gut microbiota composition.

**Materials and methods:**

GPs were extracted via hot water extraction and ethanol precipitation. Seventy-two SPF-grade male mice were randomly divided into six groups and treated with different doses of GPs or *Astragalus* polysaccharides (APS), followed by PRV challenge. Clinical parameters, inflammatory cytokines (TNF-*α*, IL-6, IL-4, IL-10), oxidative stress markers (SOD, CAT, MDA), histopathology, tight junction protein expression (Occludin, ZO-1), sIgA levels, intestinal permeability, viral load, and gut microbiota profiles were assessed.

**Results:**

GP administration significantly alleviated PRV-induced symptoms, reduced mortality and disease activity index, and improved food intake. Medium and high doses notably downregulated TNF-*α* and IL-6, while upregulating IL-4 and IL-10. Antioxidant activities (SOD, CAT) were enhanced, and MDA levels were decreased. Histological analyses showed recovery from villus atrophy and goblet cell loss. GPs improved tight junction integrity, elevated sIgA, reduced gut permeability and viral burden. Microbiota analysis revealed increased *α*-diversity, enrichment of *Lactobacillus* and *Bacteroides*, and suppression of potential pathogens. Functional predictions suggested GPs influenced immunity- and metabolism-related microbial pathways.

**Conclusion:**

GPs exert protective effects against PRV-induced intestinal injury by modulating immune and oxidative responses, enhancing mucosal barrier integrity, and rebalancing gut microbiota. These findings support the potential of GPs as a therapeutic agent for viral enteric diseases. To our knowledge, this is the first study to demonstrate the protective role of GPs against PRV infection *in vivo*. These findings expand current understanding of the antiviral potential of plant-derived polysaccharides and highlight GPs as a promising candidate for the development of novel polysaccharide-based therapeutics for viral enteric diseases.

## 1 Introduction

Pseudorabies (PR), also known as Aujeszky’s disease, is a highly contagious disease caused by the pseudorabies virus (PRV) (1). PRV is a globally distributed virus that primarily infects pigs, cattle, sheep, mice, and rabbits, among other animals. Among these, pigs serve as the reservoir host, and both infected and virus-carrying pigs are major sources of transmission. The virus is also capable of infecting various wild and carnivorous animals. Clinically, the infection is characterized by distinctive symptoms, most notably intense pruritus (itching) (2). Research shows that PRV infection can cause necrotizing enteritis and disrupt the gut microbiota, ultimately leading to intestinal homeostasis imbalance. Changes in the intestinal environment further exacerbate the destruction of the microbiota, resulting in increased inflammation (3). Currently, attenuated live vaccines, such as Bartha K61, are widely used worldwide to prevent PRV infection in pigs. However, increasing evidence shows that these vaccines do not provide complete protection against the new PRV variants that have emerged since the end of 2011 (4). Therefore, identifying diverse and effective therapeutic approaches has become a crucial strategy to address the threats posed by PRV variants.

Natural products, particularly plant-derived polysaccharides, have attracted increasing attention as alternative or complementary antiviral strategies due to their immunomodulatory and gut microbiota-regulating properties (5). Notably, the use of traditional Chinese medicine polysaccharides for the treatment of PRV-induced viral enteritis represents a highly innovative approach, highlighting their potential as novel therapeutic agents in this context. Among them, *Glycyrrhiza* (*Glycyrrhiza* spp.), an important medicinal herb in traditional Chinese medicine, represents a promising candidate. Polysaccharides are one of the main bioactive components of *Glycyrrhiza*, Studies have shown that *Glycyrrhiza* Polysaccharides (GPs) has biological activities such as immune regulation (6) and antioxidant (7), antitumor (8), antiviral (9), and antibacterial (10) properties. GPs effectively alleviates LPS-induced acute colitis and promotes gut health by downregulating inflammatory cytokines (such as TNF-*α*, IL-1β, and IL-6), enhancing the expression of tight junction proteins, improving intestinal barrier function, and modulating gut microbiota composition by increasing beneficial bacteria (such as *Lactobacillus*, *Bacteroides*, and *Akkermansia*) while suppressing pathogenic bacteria (11). Wangdi Song (12) found that low molecular weight GPs effectively alleviates cyclophosphamide-induced intestinal barrier damage and gut microbiota imbalance by repairing intestinal structure, increasing goblet cell number and mucus secretion, improving Th1/Th2 balance, and enhancing levels of short-chain fatty acids (SCFAs) and beneficial gut bacteria. However, its mechanism of action in viral enteritis, especially in models induced by PRV, remains unclear. Previous studies have demonstrated that plant-derived extracts can also exert prebiotic effects by modulating the gut microbiota (13, 14). For example, water extract from *Abies alba* wood has been shown to promote the growth of *Lactobacillus paracasei*, *L. acidophilus*, *L. rhamnosus*, *L. gasseri*, *L. crispatus*, and *L. bulgaricus* without affecting lignan metabolism, suggesting that plant-derived bioactive compounds may exert health benefits by improving the gut microbial environment (15). *Coix seed* polysaccharide (CSP) alleviates DSS-induced ulcerative colitis by reducing weight loss, lowering inflammatory cytokines, restoring intestinal barrier integrity, and modulating gut microbiota and beneficial metabolites such as 3-hydroxybutyrate (16). However, despite these advances, direct evidence linking PRV-induced enteritis with TCM interventions, particularly GPs, remains limited. Specifically, it is unclear whether GPs can directly mitigate PRV-induced intestinal injury by modulating immune responses, oxidative stress, mucosal barrier integrity, and gut microbiota composition.

This study aims to evaluate the protective effects and underlying mechanisms of GPs against PRV-induced infection in mice, with a focus on its therapeutic potential in viral enteritis. Specifically, the study seeks to determine whether GPs exerts its effects through modulation of immune responses, attenuation of oxidative stress, restoration of intestinal barrier integrity, and regulation of gut microbiota. The findings are expected to provide a theoretical foundation for the development and application of traditional Chinese medicine polysaccharides in the prevention and treatment of viral intestinal diseases.

## 2 Materials and methods

### 2.1 Materials and reagents

The *Glycyrrhiza* used in this study was purchased from the Yunnan Traditional Chinese Medicine Market and identified as *Glycyrrhiza uralensis* roots by the Department of Veterinary Traditional Chinese Medicine at Yunnan Agricultural University. Seventy-two SPF-grade male Kunming mice (5–6 weeks old, 22 ± 3 g) were obtained from Yunnan University (license number SCXK (Dian) K2015-0002). The PRV-XD-F3 virus strain was supplied by the Yunnan Academy of Animal Science and Veterinary Medicine.

### 2.2 Extraction and purification of polysaccharides

GPs were extracted using a water extraction–alcohol precipitation method. Briefly, 500 g of *Glycyrrhiza* root powder was refluxed with 95% ethanol at 60 °C under reduced pressure three times (4 h each) to remove pigments and lipid-soluble impurities. The dried residue was used for polysaccharide extraction. The residue was mixed with distilled water at a material-to-liquid ratio of 1:25, subjected to ultrasonic treatment at 70 °C (300 W, 20 min), and subsequently extracted at 75 °C for 6 h. The extract was filtered, concentrated to 300 mL, cooled, and precipitated with 1,500 mL of 95% ethanol for 24 h. The precipitate obtained after centrifugation (4,000 rpm, 10 min) was collected as crude GPs and stored at −20 °C. Crude polysaccharides were deproteinized using the Sevag method (chloroform:n-butanol = 4:1), concentrated under reduced pressure, and freeze-dried at −80 °C for 72 h to yield purified GPs powder. Polysaccharide content was determined by the phenol–sulfuric acid method using a glucose calibration curve.

### 2.3 Infection of PRV in BHK-21 cells and determination of viral titer

BHK-21 cells were cultured in DMEM supplemented with 10% fetal bovine serum and antibiotics (100 μg/mL streptomycin and 100 IU/mL penicillin) at 37 °C in a 5% CO₂ incubator. One milliliter of PRV viral stock was inoculated onto confluent BHK-21 cells and allowed to adsorb for 2 h. After removal of the viral inoculum, maintenance medium was added for continued culture until approximately 90% cytopathic effect (CPE) was observed. The infected cells were subjected to three freeze–thaw cycles at −80 °C, followed by centrifugation at 12,000 rpm for 15 min at 4 °C. The supernatant containing virus was aliquoted and stored at −80 °C. For TCID₅₀ determination, the virus stock was serially diluted 10-fold in maintenance medium and inoculated onto BHK-21 cells in 96-well plates (100 μL per well) with eight replicates per dilution and blank controls. CPE was monitored, and the TCID₅₀ was calculated using the Reed-Muench method.

### 2.4 Animal feeding and model establishment

**Figure 1:**
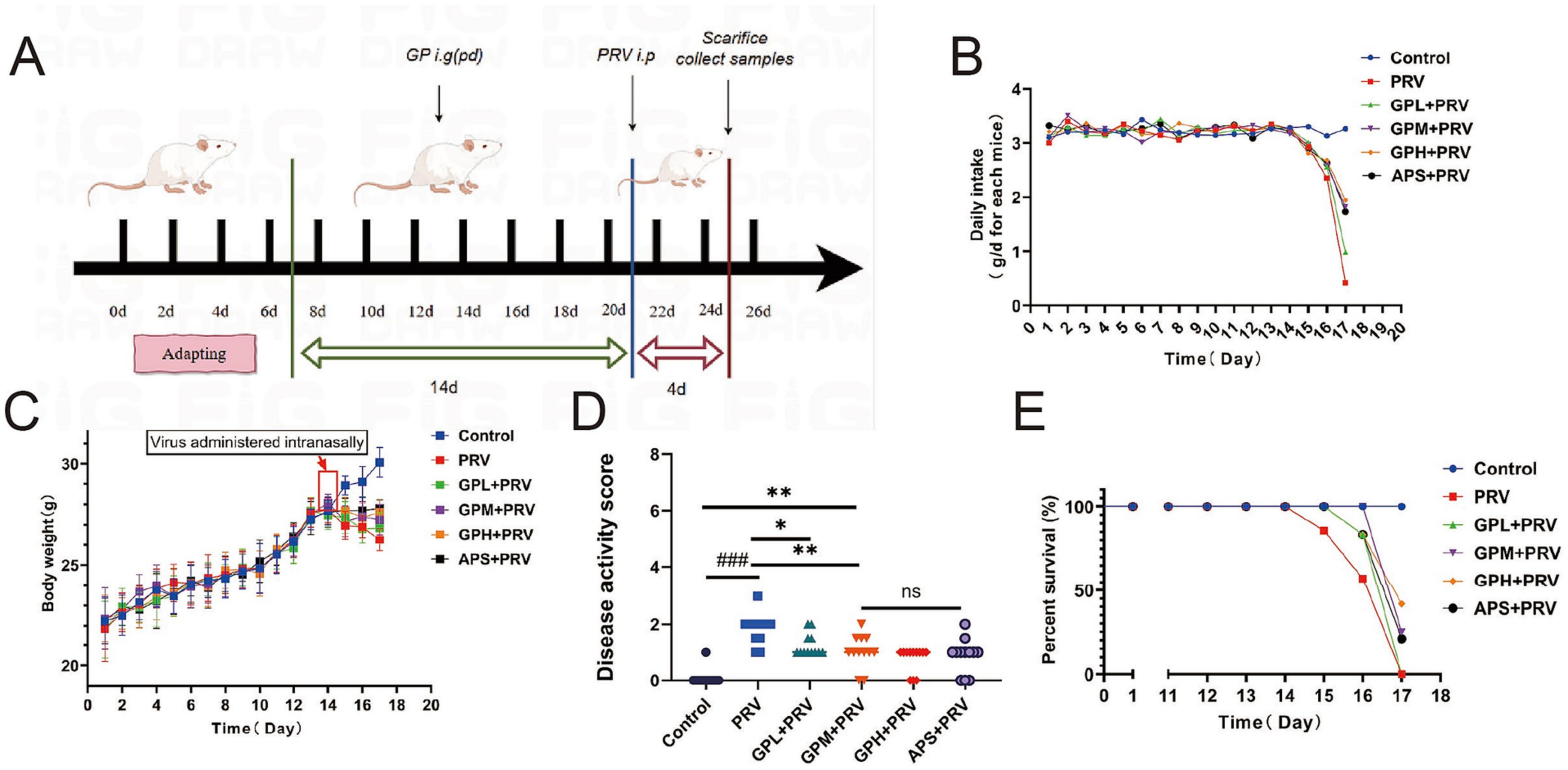
GPs alleviate clinical symptoms and improve survival in PRV-infected mice. **(A)** Experimental design. **(B)** Daily food intake per cage to per animal. **(C)** Body weight changes over time. Body weight gain was suppressed in the PRV group, whereas GPs treatment improved weight maintenance. **(D)** Disease activity index (DAI) score. **(E)** Survival curves. All data are presented as mean ± SD (*n* = 12)*. ###p* < 0.001 vs. control group; *p* < 0.05, *p < 0.01 vs. PRV group; ns, not significant.

Seventy-two SPF-grade, 8-week-old male Kunming mice were acclimated for 1 week and randomly divided into six groups (n = 12): normal control (CON), PRV infection model (PRV), high-dose GPs (GPH, 400 mg/kg), medium-dose GPs (GPM, 200 mg/kg), low-dose GPs (GPL, 100 mg/kg), and positive control with APS (200 mg/kg) (11). The treatment groups received oral administration for 14 consecutive days. Subsequently, PRV infection was induced by intraperitoneal injection of 30 μL PRV suspension (10^5.6^ TCID₅₀/0.1 mL) (Figure 1A). Body weight, food intake, and clinical symptoms were monitored daily. On day 3 post-infection, mice were euthanized by CO₂ asphyxiation in accordance with AVMA guidelines, and death was confirmed before tissue collection. Mortality was recorded, and survival curves were generated. Blood samples were collected for serum separation. The colon was dissected and measured, while colon contents, duodenum, and colon segments (~5 cm) were harvested. Portions of tissues were fixed in 4% paraformaldehyde, mucosa was scraped for centrifugation, and the remaining tissues were wrapped in foil. All samples were snap-frozen in liquid nitrogen and stored at −80 °C for subsequent analyses.

### 2.5 Disease activity index scoring

Starting from the establishment of the PRV infection model, the mice were assessed daily at fixed times for mental state, motor ability, food intake, and fur condition, and their body weight was measured. Stool characteristics and occult blood were also observed. The Disease Activity Index (DAI) was calculated by summing the scores of percentage body weight loss, stool consistency, and presence of occult blood to comprehensively reflect the severity of disease activity. The scoring criteria were as follows: no weight change, normal stool, and no occult blood scored 0 points; 1–5% weight loss, softer stool, and light blue occult blood scored 1 point; 6–10% weight loss, loose stool, and obvious blue occult blood scored 2 points; 11–15% weight loss, mucus-like stool, and large amounts of dark blue occult blood scored 3 points; weight loss over 15%, watery stool, and visible blood in stool scored 4 points. The DAI scoring was performed in a blinded manner by two independent observers to ensure objectivity and reliability. A total of *n* = 12 mice were included in each group.

### 2.6 Enzyme-linked immunosorbent assay

The levels of TNF-*α*, IL-6, IL-4, and IL-10 in mouse serum were measured using enzyme-linked immunosorbent assay (ELISA) according to the instructions provided with the ELISA kits (batch numbers: Ml002095, Ml002293, Ml002149, Ml037873) from Shanghai Enzyme-linked Biotechnology Co., Ltd. Serum levels of superoxide dismutase (SOD), catalase (CAT), and malondialdehyde (MDA) were also detected by ELISA using corresponding kits (batch numbers: Ml001998, Ml037752, Ml002001) from the same company. All experiments were conducted under strictly controlled conditions to ensure data accuracy and reproducibility.

### 2.7 Hematoxylin and eosin analysis

Duodenal and colonic tissues were fixed in 4% paraformaldehyde for 72 h, then cut into tissue blocks approximately 3 mm thick and rinsed under running water for 20 min. The tissues were dehydrated through a graded ethanol series (75, 85, 95, 100%) and cleared with xylene before being embedded in paraffin. Sections were cut at a thickness of 5 μm, floated on a 40 °C water bath, and dried. After deparaffinization using xylene and graded ethanol, the sections were stained with hematoxylin for 5 min, differentiated in acid alcohol for 3–5 s, blued, and then counterstained with eosin for 30 s. The sections were then dehydrated again through graded ethanol and xylene, and mounted with neutral resin (China National Pharmaceutical Group Chemical Reagents Co., Ltd., batch number 10004160). Microscopic observation was performed using a SOPTOP ICX41 microscope (Sunny Instrument), and images were captured. Using Image Viewer software, villus height, crypt depth, and mucosal thickness were measured to evaluate the pathological effects of PRV infection on intestinal tissue.

### 2.8 Periodic acid–Schiff analysis

Paraffin sections were baked in an oven at 65 °C for 30 min, followed by deparaffinization in xylene twice for 15 min each. The sections were then rehydrated through a graded ethanol series (100, 95, 85, 75%) and rinsed twice in distilled water for 5 min each. The sections were treated with periodic acid solution (China National Pharmaceutical Group Chemical Reagents Co., Ltd., batch number 10004160) for 8 min, rinsed under running water for 5 min, dried, and stained with Schiff’s reagent (Sigma-Aldrich, batch number S5133) in the dark for 15 min, followed by washing for 10 min. Subsequently, the sections were counterstained with hematoxylin for 2 min and blued in PBS for 3 min. The sections were dehydrated through a graded ethanol series (75, 85, 95, 100%, 5 min each) and cleared twice in xylene for 10 min each, then mounted with neutral resin. Images were captured under a microscope (Sunny Instrument, SOPTOP ICX41), and ImageJ software was used to quantitatively analyze the proportion of goblet cells in at least five fields of view in the duodenum and colon to assess changes in intestinal mucus secretion.

### 2.9 Immunofluorescence analysis

Paraffin-embedded intestinal tissue sections were first deparaffinized in xylene and rehydrated through a graded ethanol series. Antigen retrieval was performed using pH 6.0 citrate buffer in a microwave (boiled on high for 5 min, then medium heat for 3 min, followed by natural cooling). Sections were then incubated in 3% hydrogen peroxide solution for 15 min in the dark to block endogenous peroxidase activity. After blocking with 5% BSA for 15 min, the sections were incubated overnight at 4 °C in a humid chamber with primary antibodies against ZO-1 (Proteintech, Cat# 21773-1-AP) or Occludin (Proteintech, Cat# 27260-1-AP). The next day, sections were washed three times with PBS and incubated with HRP-labeled secondary antibody (Servicebio, Cat# G23303) at room temperature in the dark for 50 min. Subsequently, TYR570 fluorescent dye (red, Servicebio, Cat# G1223-570) and TYR520 fluorescent dye (green, Servicebio, Cat# G1223-520) were applied for 10 min, followed by nuclear counterstaining with DAPI (Servicebio, Cat# G1012). Finally, sections were mounted with antifade mounting medium (Servicebio, Cat# G1401) and observed under a multichannel fluorescence microscope (Nikon Eclipse C1, Japan). Fluorescence intensities of ZO-1 and Occludin were quantified using ImageJ software (NIH, USA) to compare tight junction protein expression differences among treatment groups.

### 2.10 Extraction of nucleic acids from intestinal tissue

Mouse intestinal tissues were taken from a − 80 °C freezer, and 0.1 g of tissue was weighed and ground in liquid nitrogen. Then, 25 mg of the ground tissue was mixed with 180 μL Buffer GL, 20 μL Proteinase K, and 10 μL RNase A (TaKaRa DNA Extraction Kit, Takara, Cat# D3350) and lysed in a 56 °C water bath for 10 min. Next, 200 μL Buffer GB and 200 μL of 100% ethanol were added and mixed thoroughly before transferring the mixture to a spin column and centrifuging at 12,000 rpm for 2 min. The column was washed sequentially with 500 μL Buffer WA and 700 μL Buffer WB twice. Finally, DNA was eluted with 35 μL of ddH₂O. The concentration of the extracted DNA was measured using a NanoDrop spectrophotometer and stored at −20 °C for later use.

### 2.11 Determination of intestinal viral load

Primers and TaqMan probe were designed targeting the PRV gE gene. The forward primer sequence was 5’-CTACAGCGAGAGCGACAACGA-3′, the reverse primer sequence was 5’-CGACAGCGAGCAGATGACCA-3′, and the probe sequence was 5’-HEX-CACACGGCCACGTCGCCACCTG-BHQ1-3′. The qPCR reaction system (20 μL) contained 10 μL Premix Ex Taq (Takara, Cat# RR420A), 0.5 μL each of forward and reverse primers (10 μM), 0.5 μL probe (5 μM), 2 μL DNA template, and 6.5 μL ddH₂O. PCR cycling conditions were initial denaturation at 95 °C for 3 min, followed by 40 cycles of 95 °C for 15 s, 60 °C for 45 s, and 55.3 °C for 30 s. Viral load was calculated based on the standard curve: y = −2.395x + 37.091 (R^2^ = 0.993).

### 2.12 RT-qPCR detection of intestinal tight junction protein mRNA expression

Total RNA was extracted from intestinal tissues using a Jifan fully automated nucleic acid extractor and magnetic bead kit, and cDNA was synthesized with TransScript One-Step gDNA Removal and cDNA Synthesis SuperMix (TransGen Biotech, Cat# AT311). Specific primers targeting ZO-1 (forward 5’-CTGGACAGCGAAGACCACAT-3′, reverse 5’-TGCTGGTGAAGTTGGTGTTG-3′, product length 153 bp) and Occludin (forward 5’-TCACCTTGGTTCGCTCTGTC-3′, reverse 5’-AGCAGGGTGTCCTGAGAAAG-3′, product length 168 bp) were used, with *β*-actin as the internal reference gene (forward 5’-GAGATTGGCATGGCTTTATTTG-3′, reverse 5’-ACTGCTGTCACCTTCACCGTT-3′, product length 127 bp). qPCR reactions were carried out in a 25 μL system containing 12.5 μL SYBR Premix Ex Taq II (Takara, Cat# RR820A), 2 μL cDNA, 1 μL of each primer, and 8.5 μL ddH₂O. The thermal cycling protocol included initial denaturation at 95 °C for 30 s, followed by 40 cycles of 95 °C for 5 s and 60 °C for 30 s. Product specificity was confirmed by melting curve analysis, and relative expression levels of target genes were calculated using the 2^-ΔΔCt^ method.

### 2.13 Western blotting

Protein expression levels of ZO-1, Occludin, and *β*-actin were detected by Western blot. Tissue samples were lysed using RIPA lysis buffer (Beyotime, #P0013B) supplemented with PMSF (Beyotime, #ST506) and protease inhibitor cocktail (Thermo Fisher, Halt™, #78430). Tissues were ground in liquid nitrogen, and cells were scraped using a sterile cell scraper (Corning) to assist lysis. Lysates were incubated on ice and centrifuged at 12,000 rpm for 15 min at 4 °C to collect the supernatant. Protein concentration was determined using the BCA assay (Thermo Fisher Scientific, #23225): a BSA standard curve was generated, BCA working reagent was added to each well, and absorbance was measured at 562 nm using a microplate reader (BioTek, Synergy H1). Equal amounts of protein were mixed with loading buffer (5 × SDS loading buffer, Beyotime, #P0015L), denatured at 95 °C for 5 min, and resolved on SDS-PAGE gels (10% or 12%, Bio-Rad Mini-PROTEAN system). Proteins were transferred onto PVDF membranes (Millipore, Immobilon-P, #IPVH00010) using a wet transfer system (Bio-Rad, Mini Trans-Blot Cell). Membranes were blocked with 5% non-fat milk in TBST for 1 h at room temperature, followed by incubation with primary antibodies anti-ZO-1 (Proteintech, Wuhan Sanying, Cat# 21773-1-AP, rabbit, 1:1000, 230 kDa), anti-Occludin (Affinity, Cat# DF7504, rabbit, 1:1000, 59 kDa), and anti-*β*-actin (Servicebio, Cat# GB15003, rabbit, 1:5000, 42 kDa) at 4 °C overnight. After washing with TBST, membranes were incubated with HRP-conjugated secondary antibodies (Servicebio, Cat# GB23303, 1:3000) for 1 h at room temperature. Chemiluminescent signals were developed using an ECL substrate (Thermo Scientific, #32106), and images were captured with a gel imaging system (Bio-Rad, ChemiDoc MP).

### 2.14 16S rRNA gene sequencing analysis of mouse gut microbiota

To evaluate the regulatory effect of GPs on the gut microbiota of mice, colon contents from each group were collected. Microbial genomic DNA was extracted using the QIAGEN DNeasy PowerSoil Kit (QIAGEN, Cat# 12888). The V3–V4 region of the 16S rRNA gene was amplified using primers 341F (5’-CCTAYGGGRBGCASCAG-3′) and 806R (5’-GGACTACNNGGGTATCTAAT-3′) with TransStart FastPfu DNA Polymerase (TransGen Biotech, Cat# AP221). The PCR products were verified by 2% agarose gel electrophoresis and purified using the AxyPrep DNA Gel Extraction Kit (Axygen, Cat# AP-GX-500). DNA concentration was measured with Qubit 4.0 (Thermo Fisher). Libraries were constructed using the Illumina TruSeq Nano DNA LT Library Prep Kit (Cat# FC-121-4001) and, after quality control with the KAPA Library Quantification Kit (Roche, Cat# KK4824), paired-end sequencing (PE250) was performed on the Illumina NovaSeq 6,000 platform. Sequencing data were quality controlled using Cutadapt (v1.9.1) to remove low-quality and low-abundance sequences, resulting in high-quality amplicon sequence variants (ASVs). Alpha diversity (e.g., Shannon, Chao1) and beta diversity (e.g., PCoA) analyses were conducted using QIIME2 (v2020.2) to systematically assess the effects of GPs on the composition and diversity of the gut microbiota.

### 2.15 Statistical analysis

All data represent at least three independent experiments. Normally distributed data with equal variance are expressed as means ± SD and analyzed by one-way ANOVA using GraphPad Prism 10.0, with two-group comparisons by unpaired two-tailed Student’s *t*-test. Non-normal or unequal variance data were analyzed by Kruskal–Wallis test, followed by pairwise comparisons if significant. *p* < 0.05 was considered statistically significant, *p* < 0.01 highly significant, and *p* < 0.001 extremely significant. Quantitative results from immunofluorescence and PAS staining were measured using ImageJ. Additional details are provided in figure legends.

## 3 Results

### 3.1 GPs alleviate clinical symptoms and improve survival in PRV-infected mice

Table 1 shows that GPs were successfully extracted from *glycyrrhiza* powder using the Sevag method, with a yield of 3.57 ± 0.07% and a purity of 71.32 ± 1.35% (Table 2), indicating that the extraction process is simple, environmentally friendly, and cost-effective. During the gavage period (days 1–14), body weight steadily increased in all groups, with no significant differences in food intake (*p* > 0.05) (Figures 1B,C). After PRV infection, both body weight and food intake decreased in all groups except the CON, with the PRV group showing the most pronounced reduction (*p* < 0.01). On day 17, food intake in the PRV decreased by 86.8% compared to CON, whereas the decreases in the GPL, GPM, GPH, and APS groups were 69.9, 44.2, 40.5, and 46.9%, respectively. Among these, the GPM and GPH groups showed the smallest reductions, consistent with their relatively higher body weights, which were only 6.5 and 4.5% lower than CON, respectively. These results indicate that GPs alleviated PRV-induced anorexia and weight loss in a dose-dependent manner. Consistently, DAI scores were significantly elevated in the PRV group (*p* < 0.05), whereas GPs treatment markedly reduced DAI in a dose-dependent manner (Figure 1D). Mortality analysis further confirmed the protective effects: the mortality rate was 58.33% in the PRV group, which decreased to 41.67, 25.00, and 25.00% in the GPL, GPM, and GPH, respectively, and 33.33% in the APS group, while no deaths occurred in the CON (Figure 1E).

**Table 1:** Rate of GPs obtained (X ± s, *n* = 5).

Extraction batch	Initial weight of *Glycyrrhiza* (g)	Weight of GPs (g)	Extraction yield (%)	Average extraction yield	RSD (%)
1	200	7.14	3.57	3.57 ± 0.07	0.02
2	200	6.89	3.45		
3	200	7.25	3.63		
4	200	6.88	3.44		
5	200	7.13	3.57		

**Table 2:** Polysaccharide content in *Glycyrrhiza* (X ± s, *n* = 5).

Extraction batch	Polysaccharide content (%)	Average content (%)	RSD (%)
1	69.36	71.32 ± 1.35	0.02
2	73.74		
3	75.36		
4	68.57		
5	69.56		

### 3.2 GPs alleviate PRV-induced inflammation and oxidative stress in mice

**Figure 2:**
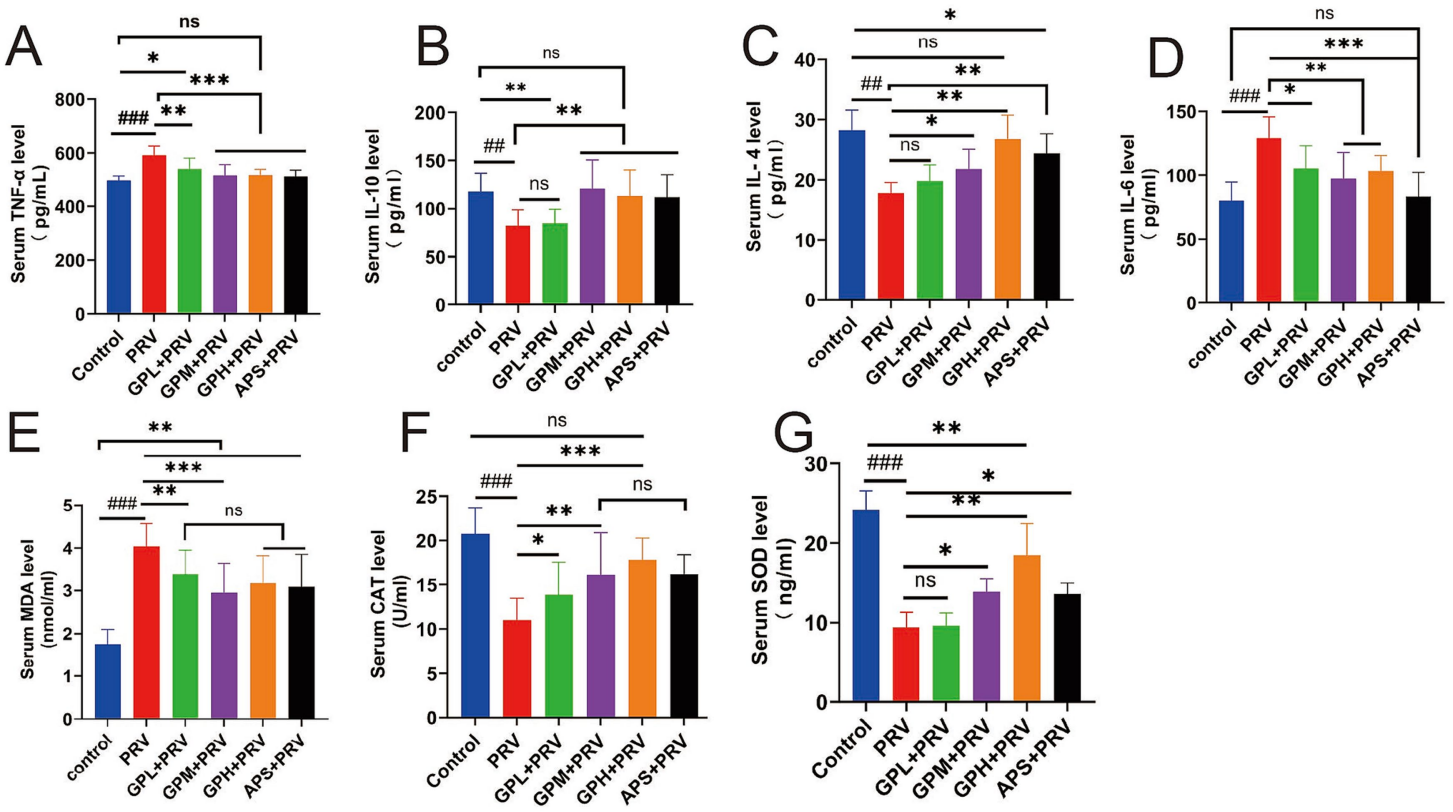
GPs alleviate PRV-induced inflammation and oxidative stress in mice. **(A–D)** Serum inflammatory cytokines: **(A)** TNF-*α*; **(B)** IL-10; **(C)** IL-4; **(D)** IL-6. **(E–G)** Serum oxidative stress markers: **(E)** MDA; **(F)** CAT; **(G)** SOD. Data are presented as mean ± SD (*n* = 12). ###*p* < 0.001, ##*p* < 0.01 vs. CON; *p* < 0.05, *p < 0.01, **p < 0.001 vs. PRV group; ns, not significant.

PRV infection markedly disrupted the balance of inflammatory cytokines and oxidative stress in mice. Compared with the CON, the PRV group exhibited significantly elevated serum TNF-*α* and IL-6 levels (*p* < 0.001) and markedly reduced IL-10 and IL-4 levels (*p* < 0.01) (Figures 2A–D). Treatment with GPs, particularly at GPM and GPH doses, significantly suppressed the elevation of pro-inflammatory cytokines and restored anti-inflammatory cytokine levels toward those observed in the CON (*p* < 0.05), indicating potent anti-inflammatory regulatory effects. Similarly, the PRV group displayed pronounced oxidative stress, as evidenced by significantly higher MDA levels (*p* < 0.001) and reduced SOD and CAT activities compared with the CON (*p* < 0.001) (Figures 2E–G). In contrast, GPs intervention significantly enhanced SOD and CAT activities (*p* < 0.01) and decreased MDA levels (*p* < 0.001) relative to the PRV group, with values approaching those of the CON. Taken together, these results demonstrate that GPs effectively alleviated PRV-induced inflammatory imbalance and oxidative stress, exhibiting a dose-dependent protective effect (Supplementary Figure 1).

### 3.3 GPs exhibit dose-dependent protective effects against small intestinal injury in PRV-infected mice

**Figure 3:**
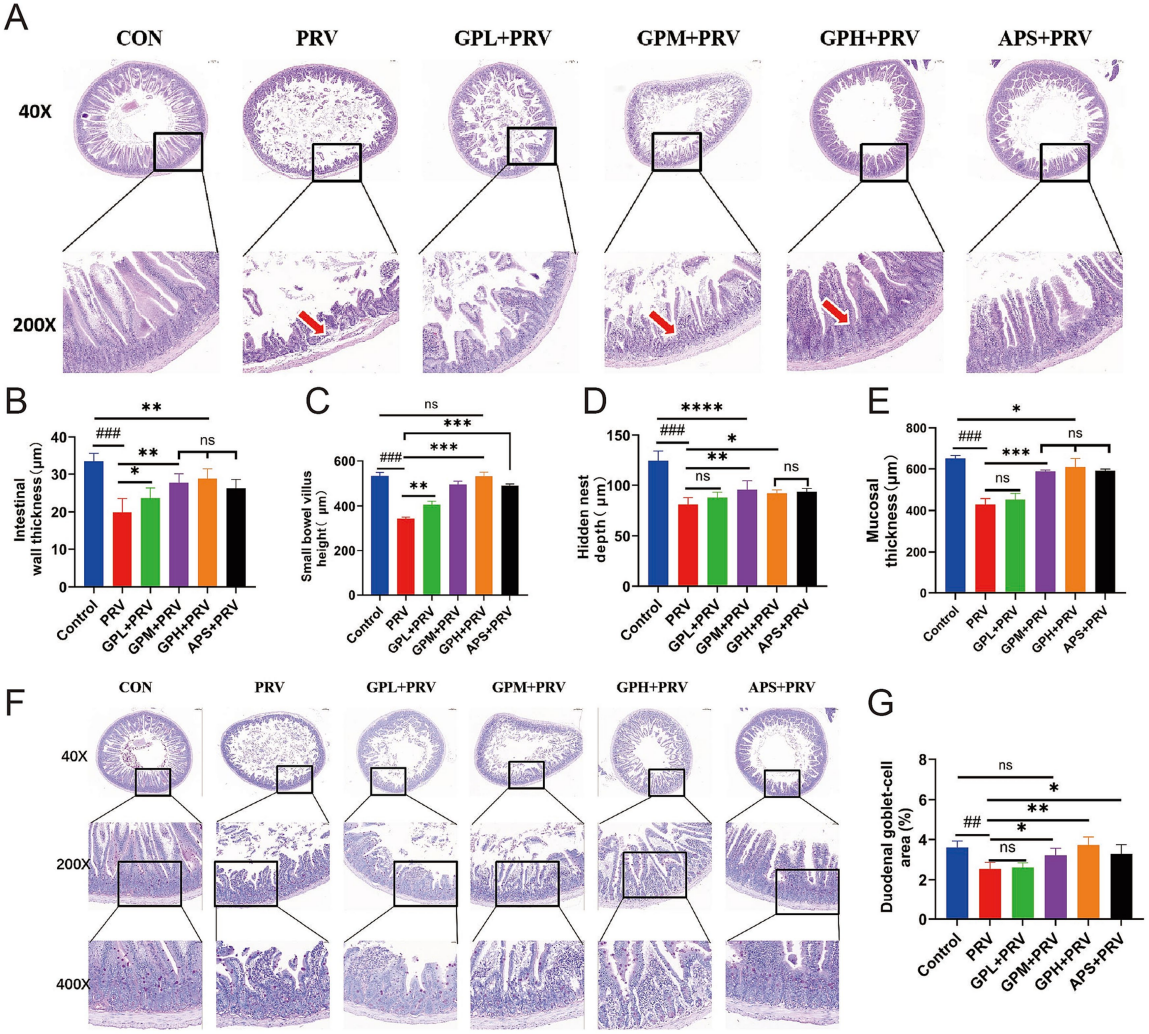
GPs exhibit dose-dependent protective effects against small intestinal injury in PRV-infected mice. **(A)** Representative H&E-stained images of the small intestine at 40 × and 200 × magnifications in CON, PRV, GPL, GPM, GPH, and APS. Red arrows indicate villus damage. **(B)** Intestinal wall thickness. **(C)** Small bowel villus height. **(D)** Hidden nest depth. **(E)** Mucosal thickness. **(F)** H&E-stained sections of the duodenum at 40×, 200×, and 400 × magnifications. **(G)** Goblet cell area in the duodenum (percentage of total tissue area). Data are presented as mean ± SD (*n* = 9). *p* < 0.001 vs. CON; *p < 0.05, **p < 0.01, ***p < 0.001 vs. PRV; ns, not significant.

Histopathological examination revealed that PRV infection caused severe intestinal injury in mice. In small intestinal sections (Figures 3A,F), marked villus atrophy, epithelial shedding, and extensive infiltration of inflammatory cells were observed. Compared with the CON, mice in the PRV group exhibited significantly reduced intestinal wall thickness (Figure 3B), small intestinal villus height (Figure 3C), crypt depth (Figure 3D), mucosal thickness (Figure 3E), and goblet cell area proportion (Figure 3G) (*p* < 0.01), indicating that PRV infection resulted in intestinal structural damage and impairment of the mucosal barrier. Relative to the PR, intervention with GPs, particularly at GPM and GPH doses, significantly ameliorated these pathological alterations. In the GPs-treated groups, villus height and mucosal thickness were markedly increased (Figures 3C,E), while crypt depth and goblet cell proportion were also significantly elevated (Figures 3D,G) (*p* < 0.05). Further comparison with the CON showed that the intestinal morphology of the GPH was closest to normal, with villus and mucosal structures nearly restored to the control level, suggesting a strong dose-dependent protective effect. The APS also exhibited partial improvement; however, its efficacy remained inferior to that of the GPH group when compared with the CON. Overall, the protective effect of GPs against PRV-induced intestinal injury exhibited a clear dose-dependent pattern (Supplementary Figure 2).

### 3.4 Dose-dependent protective effect of GPs on colonic structural damage in PRV-infected mice

**Figure 4:**
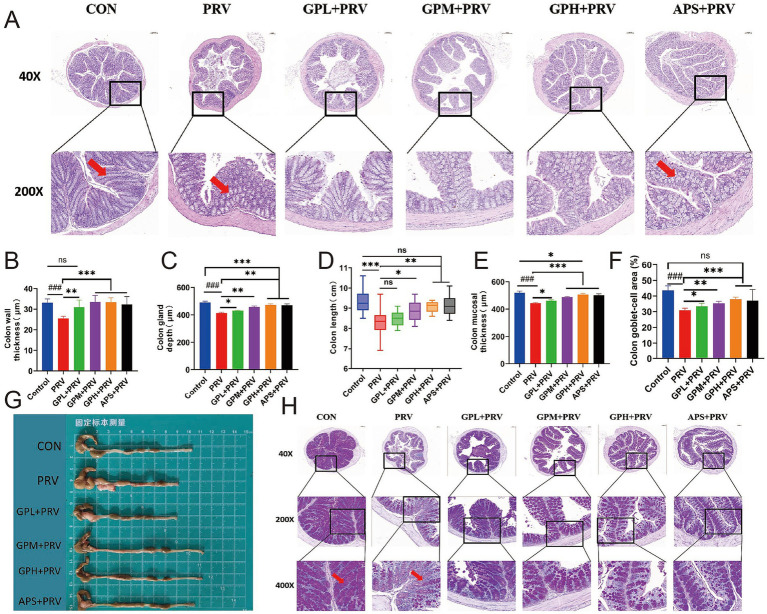
Dose-dependent Protective Effect of GPs on Colonic Structural Damage in PRV-Infected Mice. **(A)** H&E-stained colon sections at 40 × and 200 × magnifications. Red arrows indicate mucosal damage. **(B)** Colon wall thickness. **(C)** Colon gland (crypt) depth. **(D)** Colon length(cm). **(E)** Colon Mucosal thickness. **(F)** Colon Goblet cell area (% of total colon tissue). **(G)** Gross images of isolated colon tissues from each group. **(H)** H&E-stained sections at 40×, 200×, and 400 × magnifications. Red arrows indicate goblet cell loss. Data are presented as mean ± SD (*n* = 9). *p* < 0.001 vs. CON; *p < 0.05, **p < 0.01, ***p < 0.001 vs. PRV; ns, not significant.

Histopathological examination showed that PRV infection markedly damaged the colonic structure of mice. In the CON, the intestinal tissue structure was intact, with moderate intestinal wall thickness, and normal villus height and mucosal thickness (Figures 4A–C,E). In contrast, the PRV exhibited a significantly thinner intestinal wall (Figures 4B, *p* < 0.001), markedly reduced villus height (Figure 4C, *p* < 0.01), shortened colonic length (Figure 4D, *p* < 0.001), decreased mucosal thickness (Figure 4E, *p* < 0.001), and a significantly reduced goblet cell area proportion (Figures 4F,H, *p* < 0.001). In addition, villus atrophy and epithelial shedding were observed in tissue sections (Figures 4A,G, red arrows), indicating that PRV infection severely disrupted intestinal architecture and impaired mucosal barrier function. GPs intervention significantly ameliorated PRV-induced intestinal injury. The GPL group showed partial recovery in intestinal parameters, but the effects were not significant. In contrast, both the GPM and GPH exhibited significant increases in intestinal wall thickness, colonic villus height, and mucosal thickness (Figures 4B,C,E, *p* < 0.05), as well as significant improvements in colonic length and goblet cell area proportion (Figures 4D,F,H, *p* < 0.05), indicating a dose-dependent protective effect. Further comparison with the CON revealed that the intestinal morphology in the GPH was most similar to normal, with villus and mucosal structures nearly restored to control levels, and no significant differences were observed in several parameters compared with the CON (Figures 4B,D,F,G) (*p* > 0.05). In contrast, the GPM showed partial but incomplete recovery, whereas the APS exhibited limited improvement and remained significantly different from the CON, highlighting the superior protective efficacy of GPs, particularly at high doses. Moreover, gross morphological observation of the mouse intestine (Figure 4G) further supported these findings: compared with the CON, the intestines in the PRV were visibly shorter and thinner, whereas GPs treatment, especially in the GPH, partially restored intestinal length and morphology toward normal. Taken together, these results demonstrate that the protective effect of GPs against PRV-induced colonic injury was dose dependent, with the strongest efficacy observed in the GPH (Supplementary Figure 3).

### 3.5 GPs enhance the intestinal barrier by upregulating the expression of tight junction proteins

**Figure 5:**
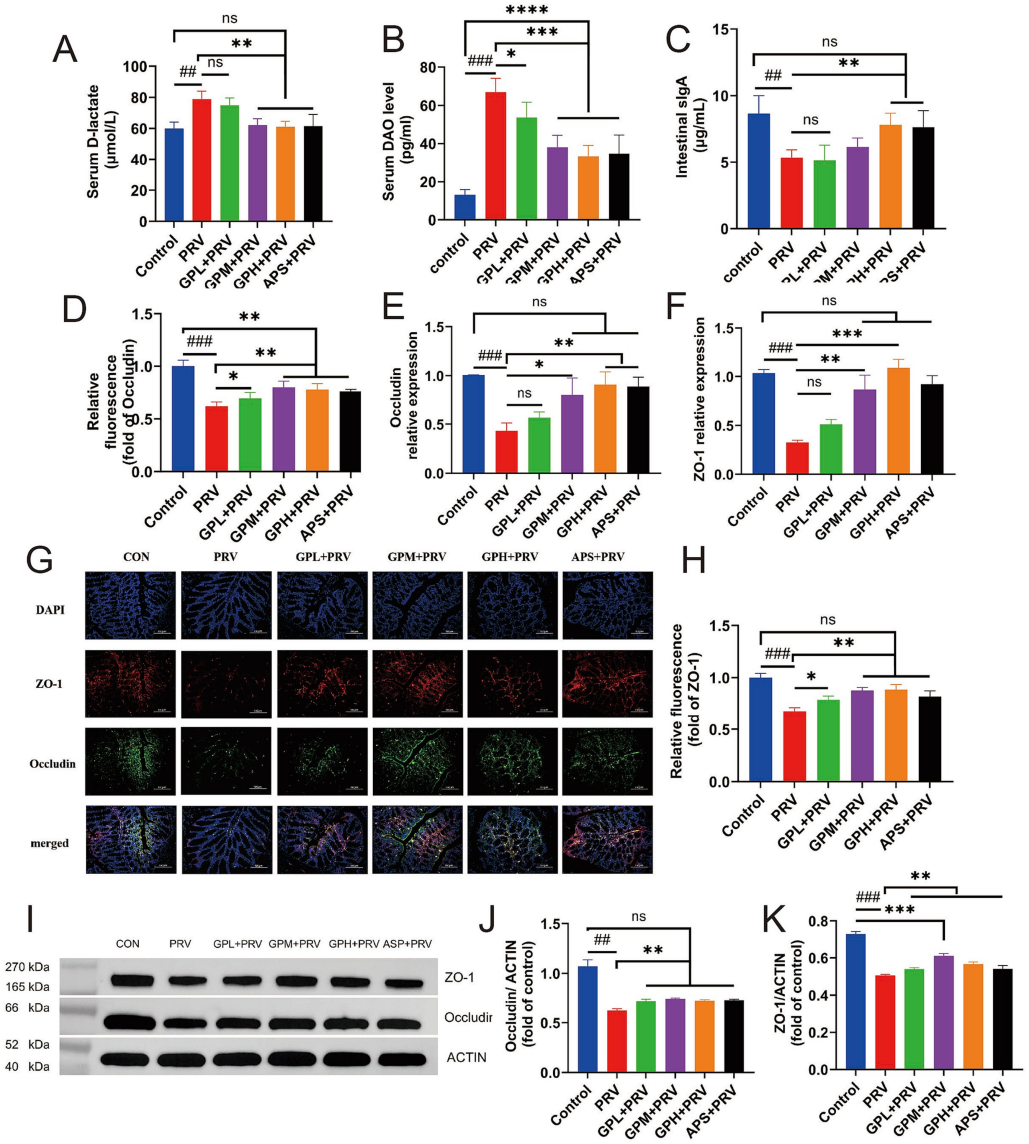
GPs enhance the intestinal barrier by upregulating the expression of tight junction proteins. **(A–C)** Serum levels of D-lactate **(A)**, DAO **(B)**, and intestinal SIgA **(C)** were measured to assess intestinal permeability and immune response (*n* = 12). **(D–F)** Relative fluorescence intensity **(D)**, mRNA expression of occludin **(E)**, and mRNA expression of ZO-1 **(F)** in intestinal tissues were evaluated. **(G)** Representative immunofluorescence images showing ZO-1 (red), occludin (green), and nuclei (DAPI, blue) in intestinal sections (*n* = 5). Scale bar = 20 μm. **(H)** Quantification of relative fluorescence intensity of ZO-1 based on immunofluorescence in **(G)** (*n* = 5). **(I)** Representative Western blot images for ZO-1 and occludin expression. ACTIN was used as a loading control. **(J,K)** Quantification of occludin **(J)** and ZO-1 **(K)** protein levels normalized to ACTIN. Data are presented as mean ± SD. *p < 0.05, **p < 0.01, ***p < 0.001 vs. PRV group; ^#^*p* < 0.05, ^##^*p* < 0.01, ^###^*p* < 0.001 vs. CON; ns, not significant.

As shown in Figure 5, PRV infection markedly impaired intestinal barrier function in mice. Compared with the CON, mice in the PRV group exhibited significantly increased intestinal permeability, with elevated serum D-lactic acid and DAO levels (Figures 5A,B, *p* < 0.01), and markedly decreased intestinal sIgA levels (Figure 5C, *p* < 0.05). In addition, the relative mRNA expression of the tight junction proteins Occludin and ZO-1 was significantly reduced (Figures 5E,F, *p* < 0.001). Consistently, immunofluorescence staining (Figures 5G,H) and Western blot analysis (Figures 5I–K) also revealed marked decreases in Occludin and ZO-1 protein levels (*p* < 0.001), indicating that PRV infection severely disrupted the intestinal tight junction structure. Compared with the CON, drug-treated groups showed partial improvement in serum D-lactic acid, DAO, sIgA, as well as Occludin and ZO-1 mRNA and protein expression levels (*p* > 0.05). In contrast, both the GPM and GPH groups, as well as the APS group, exhibited significant restoration: serum D-lactic acid and intestinal sIgA levels were nearly comparable to those of the CON (*p* > 0.05), and Occludin and ZO-1 mRNA and protein expression were markedly upregulated (*p* < 0.05), with no significant differences relative to the CON. Immunofluorescence results further showed that intestinal tight junction structures were almost restored to normal, suggesting a dose-dependent protective effect of GPs. Moreover, WB results confirmed that the APS group also showed improvement in Occludin and ZO-1 protein expression, although the extent of recovery remained inferior to that of the GPH group. Overall, while the APS group performed better than the PRV group, most parameters remained lower than those of the CON, and the protective efficacy was slightly weaker than that of GPH. Although the results of Western blot analysis (Figures 5I–K) showed weaker correlations, the overall trend remained consistent, supporting the conclusion that PRV infection severely disrupted the intestinal tight junction structure (Supplementary Figures 4, 5). Collectively, GPs intervention significantly ameliorated PRV-induced intestinal barrier damage in mice, with the GPH group showing the most pronounced protective effect and exhibiting minimal differences compared with the CON.

### 3.6 GPs modulated the composition of the gut microbiota in mice infected with PRV

**Figure 6:**
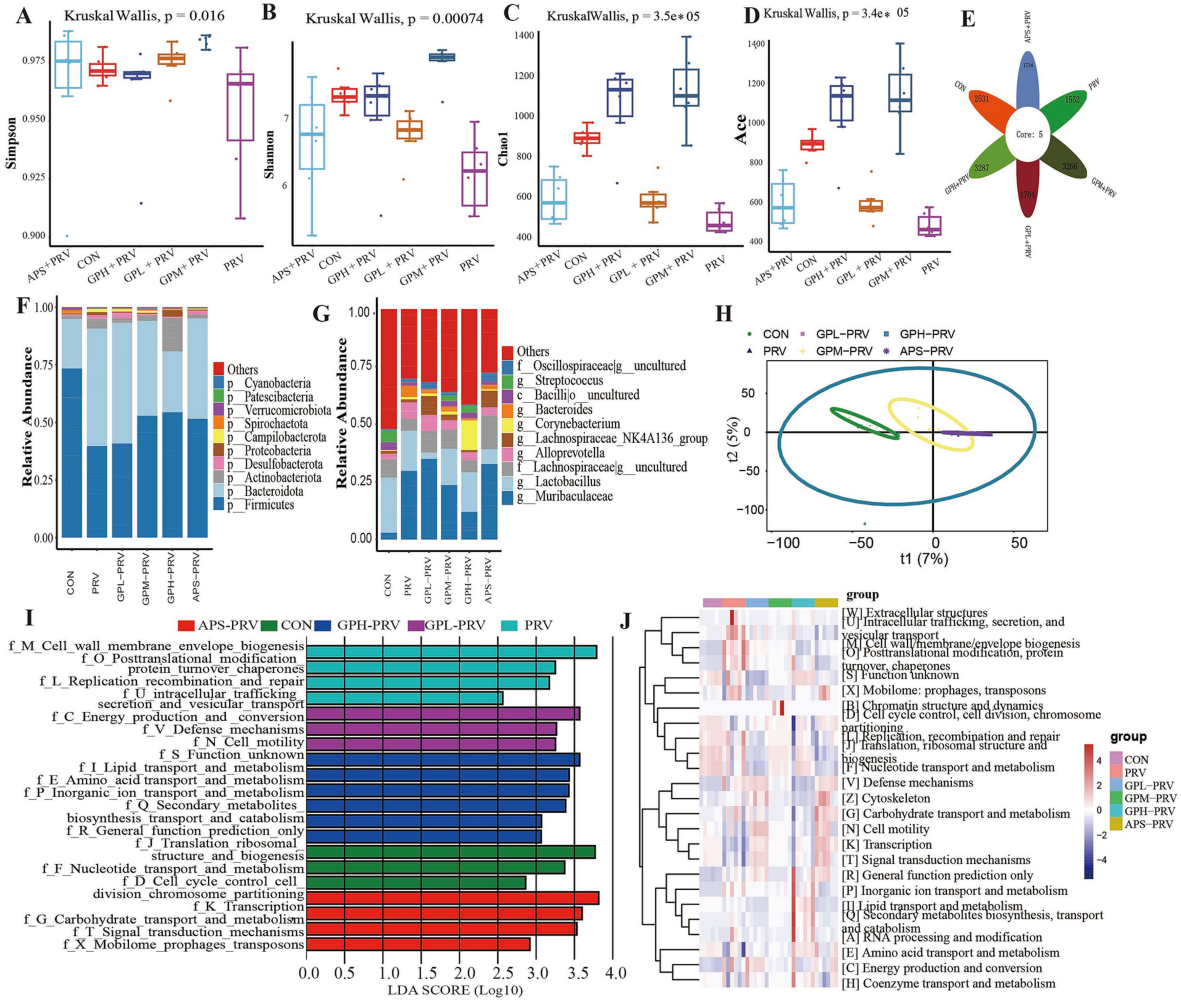
GPs modulated the composition of the gut microbiota in mice infected with PRV. **(A–D)** Alpha diversity indices: Shannon, Simpson, Chao1, ACE. (*p* = 0.00074, *p* = 0.0016, *p* = 3.5 × 10^−5^, *p* = 3.4 × 10^−5^) **(E)** Venn diagram of shared OTUs. **(F)** Relative abundance at the phylum level. **(G)** Relative abundance at the genus level. **(H)** Principal Coordinate Analysis (PCoA). **(I)** LDA scores of significantly different taxa. **(J)** Heatmap of predicted functional pathways. *n* = 6, *p* < 0.05, *p* < 0.01, *p* < 0.001; ns not significant.

PRV infection profoundly disrupted the intestinal microbiota in mice. *α*-diversity analysis (Figures 6A–D) revealed significantly reduced microbial richness and diversity, as indicated by decreased Shannon, Simpson, Chao1, and ACE indices compared with the CON group. The Venn diagram (Figure 6E) showed that PRV mice shared fewer core OTUs with CON, while GPM and GPH groups exhibited greater overlap, suggesting partial restoration of the core community. Taxonomic profiling (Figures 6F,G) demonstrated notable alterations in microbial structure, with an abnormal Firmicutes/Bacteroidetes ratio, decreased Lactobacillus and Bacteroides, and increased Prevotella in the PRV group; these changes were reversed following GPs treatment. *β*-diversity analysis (Figure 6H) further confirmed distinct clustering between PRV and CON, with microbial communities of GPM and GPH clustering more closely with the controls. LEfSe analysis (Figures 6I,J) revealed enrichment of pro-inflammatory taxa in the PRV group, whereas beneficial taxa such as Lactobacillus were enriched in GPs-treated groups. Functional prediction (Figure 6) indicated suppression of pathways related to carbohydrate, amino acid, and energy metabolism by PRV, while GPs restored these processes, particularly those involved in ribosomal activity, DNA replication, and transcription. Collectively, these findings indicate that GPs intervention effectively alleviated PRV-induced dysbiosis by reshaping microbial composition and restoring functional capacity, thereby promoting intestinal microecological balance.

## 4 Discussion

This study systematically investigated the protective effects of GPs against PRV infection in mice from multiple perspectives, including clinical symptoms, biochemical markers, intestinal histomorphology, mucosal barrier function, and gut microbiota composition. The results demonstrated that GPs significantly alleviated a series of pathophysiological changes induced by PRV infection, such as weight loss, increased mortality, cytokine storm, oxidative stress, intestinal barrier disruption, and gut microbiota dysbiosis, in a dose-dependent manner. These findings not only provide new experimental evidence for the antiviral potential of GPs but also lay a theoretical foundation for exploring the multi-target mechanisms of natural compounds in antiviral therapy.

Viral infections often trigger intense inflammatory responses, especially neurotropic viruses such as PRV, which can rapidly activate the innate immune system and induce the massive release of pro-inflammatory cytokines, resulting in a cytokine storm that causes tissue damage and even death (17). In the present study, PRV infection significantly upregulated serum levels of TNF-*α* and IL-6, indicating a strong systemic inflammatory response. Treatment with GPs led to a marked decrease in these pro-inflammatory cytokines, accompanied by increased expression of anti-inflammatory cytokines IL-4 and IL-10, suggesting that GPs exerts a regulatory effect on immune homeostasis. Previous studies have indicated that GPs can promote the release of anti-inflammatory cytokines while inhibiting the synthesis and release of pro-inflammatory mediators (12), thereby enhancing host resistance. Our *in vivo* findings further support this mechanism. Notably, the cytokine regulation pattern observed here—namely the suppression of TNF-*α*/IL-6 and the elevation of IL-4/IL-10—is consistent with earlier reports, but our data extend these findings by demonstrating that such effects also occur in the context of PRV infection, highlighting the relevance of GPs in viral neuroinflammation. Oxidative stress is a key pathological process often accompanying viral infections. PRV can induce oxidative damage by activating mitochondrial ROS production and suppressing antioxidant defense systems, leading to lipid peroxidation and apoptosis (18). In our study, PRV-infected mice exhibited significantly increased levels of malondialdehyde (MDA), alongside reduced activities of antioxidant enzymes such as SOD and CAT, indicating a classic oxidative injury state. GPs treatment effectively lowered MDA levels and restored antioxidant enzyme activities, highlighting its strong antioxidant protective potential. This antioxidant response is in line with previous findings that GPs can scavenge free radicals and enhance enzymatic defenses; however, our results demonstrate a particularly pronounced recovery of SOD and CAT activities under PRV challenge, suggesting that GPs may exert stronger protective effects in viral oxidative injury models compared to non-viral settings. Mechanistically, neurotropic viral infections such as PRV are known to activate the (19) pathway, driving the transcription of pro-inflammatory cytokines (e.g., TNF-α, IL-6) (20), while excessive ROS production further amplifies inflammation (21). At the same time, activation of the Nrf2–HO-1 pathway is a canonical defense that counteracts oxidative damage (22), and MAPK signaling (p38, JNK, ERK) integrates inflammatory and oxidative cues to regulate cytokine production (22). Prior studies have shown that GPs can suppress NF-κB activation, enhance Nrf2–HO-1 signaling, and modulate MAPK activity, thereby reducing inflammation and restoring redox balance (23, 24). In line with our findings, this suggests that GPs may protect against PRV-induced pathology by simultaneously down-regulating NF-κB, up-regulating Nrf2–HO-1, and fine-tuning MAPK signaling, ultimately breaking the vicious cycle between inflammation and oxidative stress.

Although PRV is primarily characterized by neurotropism, recent studies have shown that it can invade the central nervous system via both the blood–brain barrier (25) and the intestinal barrier (26), especially in immunocompromised hosts. As one of the body’s most crucial immune barriers, the integrity of the intestinal epithelium plays a key role in limiting systemic viral dissemination (27). In this study, PRV infection led to notable structural abnormalities in the intestinal mucosa, including villus atrophy, crypt hyperplasia, and goblet cell disorganization, indicating substantial epithelial damage. More importantly, the expression of tight junction proteins such as Occludin and ZO-1 was significantly reduced, accompanied by elevated serum levels of DAO and D-lactate, as well as reduced sIgA secretion — all suggesting increased intestinal permeability and impaired barrier function. GPs treatment significantly ameliorated both histological and molecular indicators of intestinal damage, indicating its potential to restore barrier integrity after viral injury. The mechanisms may include promoting epithelial regeneration, maintaining mucosal immune homeostasis, and upregulating tight junction protein expression. Further research is needed to elucidate the specific signaling pathways through which GPs facilitates epithelial repair, providing direction for future studies.

The gut microbiota plays a pivotal role in maintaining host immune homeostasis, and its structural diversity and functional integrity are crucial in resisting viral infections (28). Previous studies have shown that PRV infection can disrupt microbial composition, promoting the overgrowth of opportunistic pathogens and exacerbating CNS injury through the microbiota–immune–brain axis (27). In the current study, PRV infection led to reduced *α*-diversity of gut microbiota, with a decline in beneficial bacteria such as *Lactobacillus*, and an increased abundance of potential pathogens such as *Prevotella* and *Staphylococcus*, indicating significant dysbiosis. GPs intervention notably restored microbial diversity and increased the relative abundance of beneficial bacteria, suggesting a strong probiotic effect. Importantly, these microbial shifts are functionally relevant to host immunity. For instance, *Lactobacillus* and *Bacteroides* are major producers of SCFAs, which can suppress pro-inflammatory cytokine production and promote regulatory T cell differentiation (29–31), thereby alleviating excessive inflammation. In contrast, *Prevotella* enrichment has been associated with Th17 cell activation and chronic inflammation, potentially aggravating viral pathogenesis (32, 33). Further PICRUSt functional prediction analysis showed that GPs treatment restored pathways related to fatty acid synthesis and amino acid metabolism. This may indirectly regulate host immunity and barrier function by modulating the profile of metabolic products such as SCFAs and tryptophan metabolites (34). It should be emphasized, however, that these findings regarding SCFAs functions were primarily based on PICRUSt functional predictions rather than direct observations of typical SCFA-producing taxa. This limitation highlights the need for future studies combining targeted metabolomics with inflammatory and oxidative markers to validate the proposed “microbiota–metabolite–immune” axis. Taken together, these effects suggest that GPs may maintain intestinal homeostasis via the microbiota–metabolite axis, supporting its potential as a functional prebiotic.

Despite the comprehensive insights gained in this study, some limitations remain. Firstly, the focus was mainly on *in vivo* observations, without thoroughly investigating whether GPs directly interferes with the PRV replication cycle. Future studies should integrate virological assays, such as viral titers and protein expression analyses, to evaluate the direct antiviral potential of GPs. Secondly, the current model utilized mice, which differ immunologically from pigs, the natural host of PRV. Further validation in pig models is essential to assess the efficacy and safety of GPs in a clinically relevant context. In addition, more translational barriers should be considered, including the oral bioavailability, metabolic stability, and practical feasibility of GPs administration in real-world settings. Finally, GPs is a complex mixture of polysaccharides with undefined active components. Future studies should aim to identify its key functional monomers or structural domains through structure–activity relationship analyses, laying a foundation for the development of standardized antiviral therapeutics. Given that PRV primarily threatens pigs, it is also worth discussing the potential application of GPs in veterinary practice, for instance as a feed additive for preventive intervention, to enhance the practical value of these findings.

## 5 Conclusion

GPs treatment significantly reduced PRV-induced mortality, alleviated clinical symptoms, and improved intestinal morphology in infected mice. These protective effects were associated with downregulation of pro-inflammatory cytokines (TNF-*α*, IL-6), upregulation of anti-inflammatory cytokines (IL-4, IL-10), enhancement of antioxidant enzyme activities (SOD, CAT), restoration of tight junction proteins (Occludin, ZO-1), and elevation of mucosal sIgA levels. Moreover, GPs decreased viral burden and reshaped gut microbiota by increasing beneficial genera such as *Lactobacillus* and *Bacteroides* while suppressing potential pathogens. Collectively, these findings provide mechanistic evidence that GPs protect against PRV-induced intestinal injury through coordinated regulation of immunity, oxidative stress, barrier integrity, and microbial homeostasis, supporting their potential as a natural therapeutic agent for viral enteric diseases.

## Data Availability

The sequencing data generated in this study have been deposited in the Genome Sequence Archive (GSA) under accession number CRA027930, and are publicly available at https://ngdc.cncb.ac.cn/gsa/browse/CRA027930.

## Glossary

GPs: *Glycyrrhiza* Polysaccharides
PRV: Pseudorabies Virus
APS: *Astragalus* Polysaccharides
TNF-α: Tumor Necrosis Factor-alpha
IL-6: Interleukin-6
IL-4: Interleukin-4
IL-10: Interleukin-10
SOD: Superoxide Dismutase
CAT: Catalase
MDA: Malondialdehyde
ZO-1: Zonula Occludens-1
sIgA: Secretory Immunoglobulin A
DAO: Diamine Oxidase
DAI: Disease Activity Index
qPCR: Quantitative Polymerase Chain Reaction
RT-qPCR: Reverse Transcription qPCR
BHK-21: Baby Hamster Kidney-21
SPF: Specific Pathogen Free
CPE: Cytopathic Effect
TCID₅₀: 50% Tissue Culture Infectious Dose
ELISA: Enzyme-Linked Immunosorbent Assay
H&E: Hematoxylin and Eosin
PAS: Periodic Acid-Schiff
HRP: Horseradish Peroxidase
DAPI: 4′,6-diamidino-2-phenylindole
PVDF: Polyvinylidene Fluoride
BCA: Bicinchoninic Acid
SDS-PAGE: Sodium Dodecyl Sulfate Polyacrylamide Gel Electrophoresis
ECL: Enhanced Chemiluminescence
ASV: Amplicon Sequence Variant
OTU: Operational Taxonomic Unit
LEfSe: Linear Discriminant Analysis Effect Size
PCoA: Principal Coordinate Analysis
NMDS: Non-metric Multidimensional Scaling
PICRUSt: Phylogenetic Investigation of Communities by Reconstruction of Unobserved States
KEGG: Kyoto Encyclopedia of Genes and Genomes.
