# Supplementary material for: Therapeutic potential of Glycyrrhiza polysaccharides in pseudorabies virus infection: immune modulation, antioxidant activity, and gut microbiota restoration
Source: Front Vet Sci. 2025 Oct 17;12:1679013. doi: 10.3389/fvets.2025.1679013 (PMC12575151; doi:10.3389/fvets.2025.1679013)
Supplement: Supplementary file 1 [file Data_Sheet_1.pdf]

Supplementary Material

1.1 Supplementary Figures

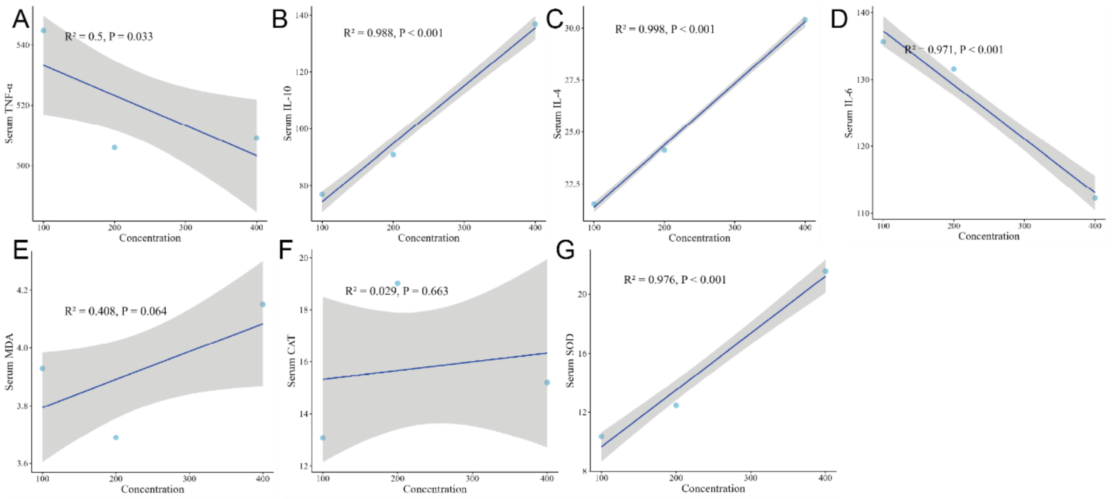

Supplementary Figure 1. Correlation analysis between Glycyrrhiza polysaccharide dose and serum inflammatory cytokines and antioxidant markers in mice.
(A) TNF-α; (B) IL-10; (C) IL-4; (D) IL-6; (E) MDA; (F) CAT; (G) SOD.

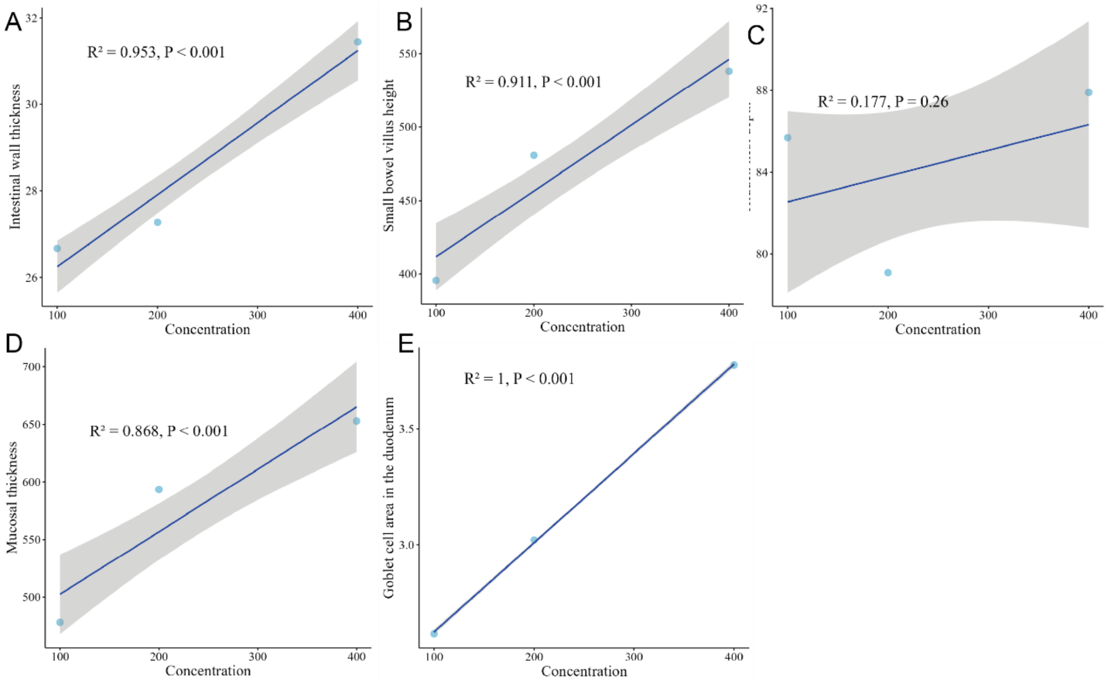

Supplementary Figure 2. Correlation analysis between Glycyrrhiza polysaccharide dose and intestinal morphological parameters in mice.

(A) Intestinal wall thickness; (B) Small bowel villus height; (C) Crypt depth; (D) Mucosal thickness; (E) Goblet cell area in the duodenum.

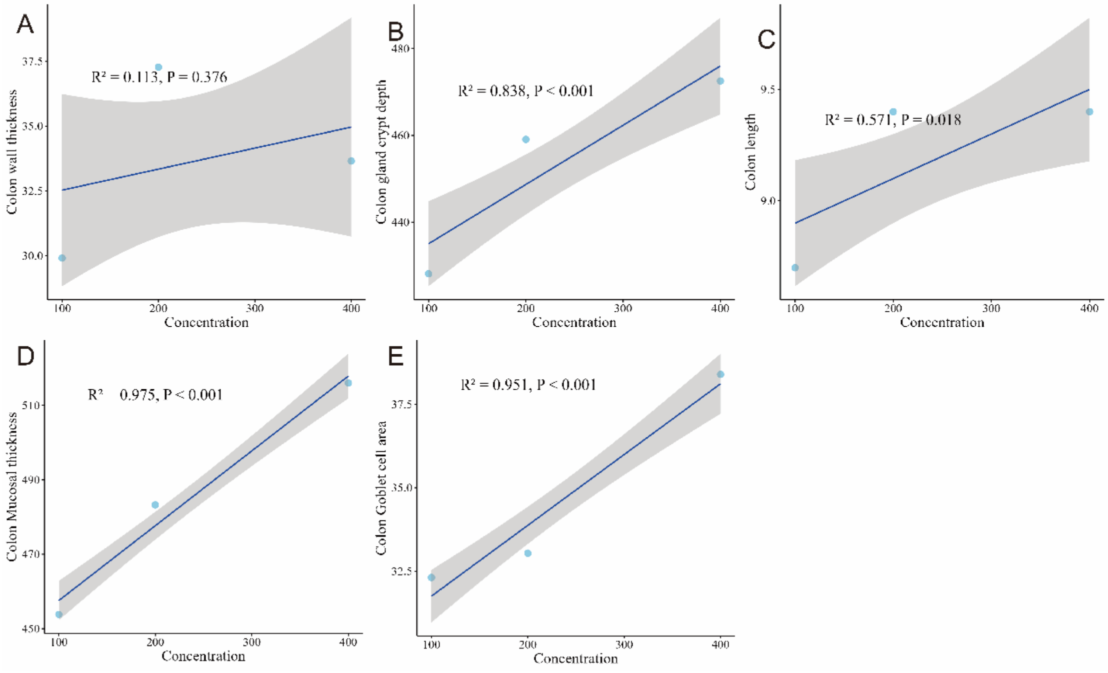

Supplementary Figure 3. Correlation analysis between Glycyrrhiza polysaccharide dose and colonic histological parameters in mice.
(A) Colon wall thickness; (B) Colon gland crypt depth; (C) Colon length; (D) Colon mucosal thickness; (E) Colon goblet cell area.

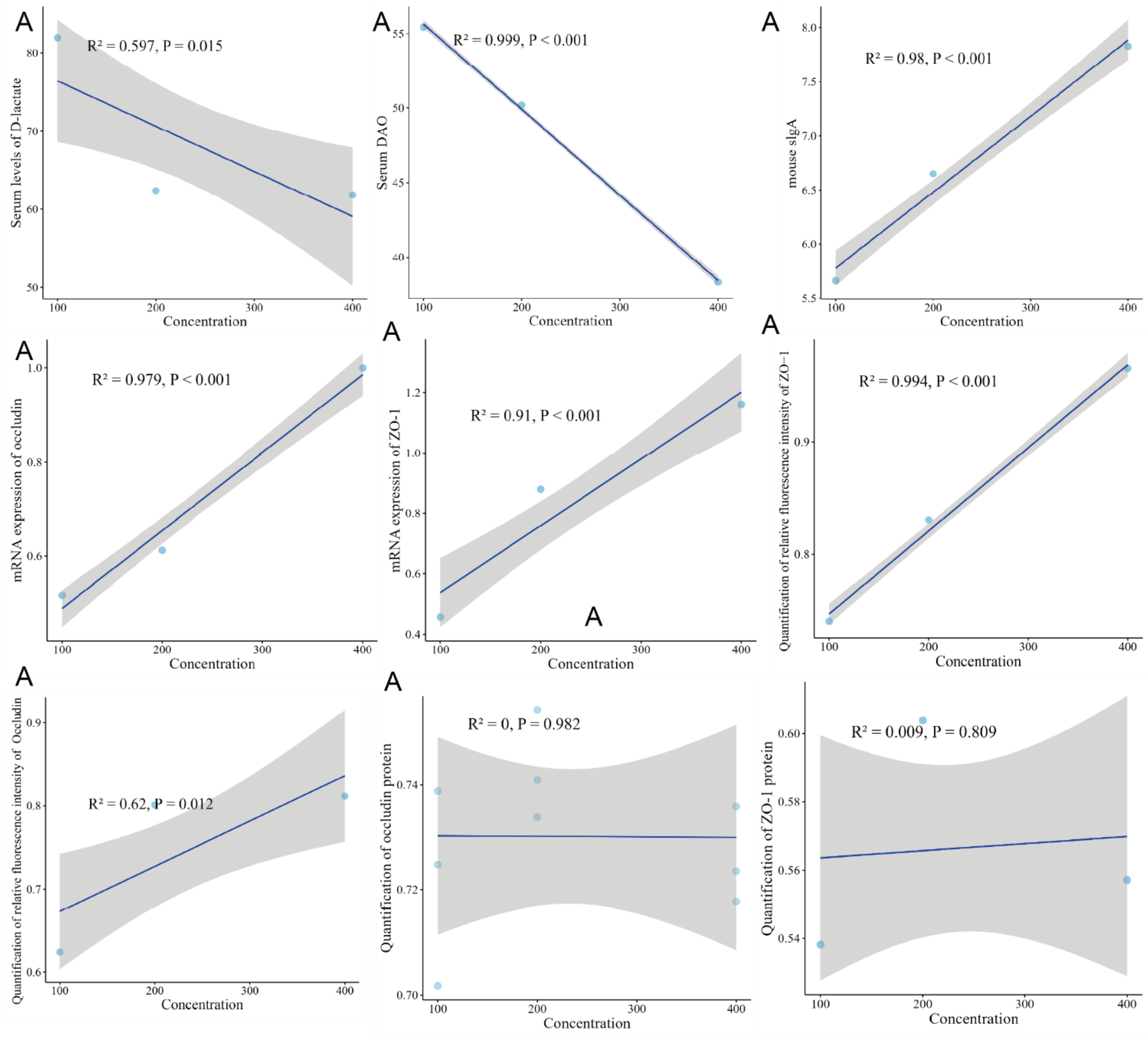

Supplementary Figure 4. Regression analysis of metabolite concentration with intestinal barrier and serum indicators.
(A) Serum D-lactate levels; (B) Serum DAO levels; (C) Fecal sIgA levels; (D) mRNA expression of Occludin; (E) mRNA expression of ZO-1; (F) Relative fluorescence intensity of ZO-1; (G) Relative fluorescence intensity of Occludin; (H) Quantification of Occludin protein; (I) Quantification of ZO-1 protein.

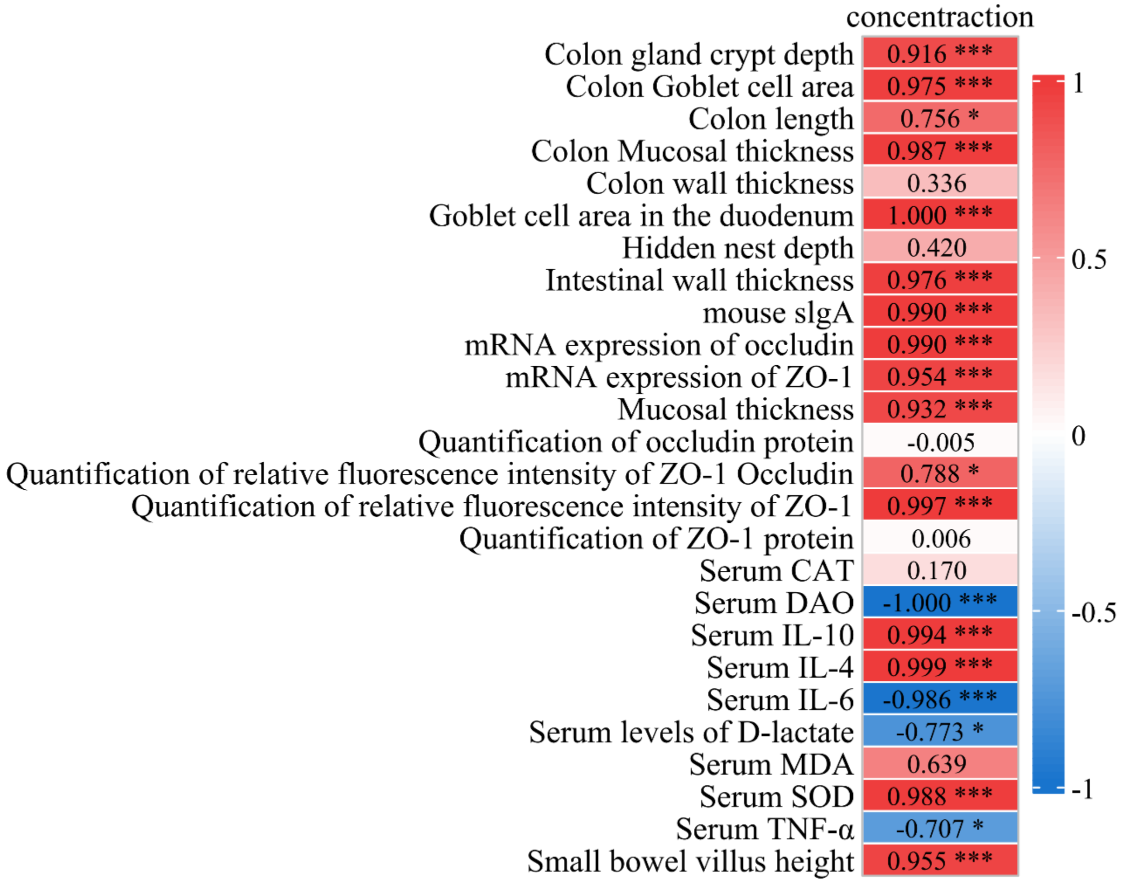

Supplementary Figure 5. Correlation heatmap between metabolite concentration and intestinal barrier function and serum indicators.
The heatmap depicts correlations between metabolite concentration and various histological parameters, intestinal barrier-related molecules, and serum inflammatory factors. Red indicates positive correlations, blue indicates negative correlations, and values represent correlation coefficients (r). Asterisks denote statistical significance (*$p < 0.05$, **$p < 0.01$, ***$p < 0.001$). Results show that metabolite concentration was significantly positively correlated with colon crypt depth, goblet cell area, mucosal thickness, tight junction molecules (Occludin, ZO-1) mRNA expression, and sIgA levels, while showing significant negative correlations with serum DAO, IL-6, and TNF-α level.
